# Possible Synergistic Effects of Glutathione and C-Reactive Protein in the Progression of Liver Cirrhosis

**DOI:** 10.3390/nu10060678

**Published:** 2018-05-27

**Authors:** Chia-Yu Lai, Shao-Bin Cheng, Teng-Yu Lee, Hsiao-Tien Liu, Shih-Chien Huang, Yi-Chia Huang

**Affiliations:** 1 Division of General Surgery, Department of Surgery, Taichung Veterans General Hospital, Taichung 40705, Taiwan; chiayulai@vghtc.gov.tw (C.-Y.L.); sbc@vghtc.gov.tw (S.-B.C.); langhsky1981@gmail.com (H.-T.L.); 2 Graduate Program in Nutrition, Chung Shan Medical University, Taichung 40201, Taiwan; 3 School of Medicine, Chung Shan Medical University, Taichung 40201, Taiwan; tylee@vghtc.gov.tw; 4 Division of Gastroenterology and Hepatology, Taichung Veterans General Hospital, Taichung 40705, Taiwan; 5 Department of Nutrition, Chung Shan Medical University, Taichung 40201, Taiwan; chienchien2011@gmail.com; 6 Department of Nutrition, Chung Shan Medical University Hospital, Taichung 40201, Taiwan

**Keywords:** C-reactive protein, neutrophil-to-lymphocyte ratio, glutathione, Child–Turcotte–Pugh score, liver cirrhosis

## Abstract

Liver cirrhosis is often associated with increased inflammatory responses and changes of glutathione (GSH) status. The possible interactions between these two factors in mediating damages of liver function remain unclear. Here, we measured the inflammatory responses and GSH status in liver cirrhotic patients and compared them with healthy subjects. In addition, we assessed the relationship of the GSH status and levels of inflammatory markers with the severity of the disease. This was a cross-sectional study. In total, we recruited 63 liver cirrhotic patients with Child–Turcotte–Pugh class A scores, and 12 patients with class B–C scores, together with 110 healthy subjects. Patients with class B–C scores showed the highest level of high-sensitivity C-reactive protein (hs-CRP) when compared with class A patients or healthy subjects. Patients in class A group had significantly higher GSH levels when compared with class B–C group or healthy subjects. After adjusting for potential confounders and each other, serum hs-CRP levels showed positive association with the Child–Turcotte–Pugh scores, while GSH levels showed negative association with Child–Turcotte–Pugh scores. Interactions were found between levels of plasma GSH and serum hs-CRP (β = 0.004, *p* = 0.016). CRP and GSH levels, which had showed interactions, were associated with the severity of liver cirrhosis.

## 1. Introduction

The end stage of liver fibrosis is liver cirrhosis, which involves the loss of liver cells and the formation of irreversible scarring. It is the 10th leading cause of death among adults in Taiwan (Health Promotion Administration, Ministry of Health and Welfare, Taiwan, 2016). The progression of liver cirrhosis is a multifactorial process, in which inflammation plays an important role. Such cirrhotic patients have elevated systemic inflammatory status [1,2,3,4]. Several markers of systemic inflammatory responses, such as C-reactive protein and neutrophil-to-lymphocyte ratio (NLR), are both inexpensive and easy-to-measure systemic inflammatory markers, and likely to predict outcomes in cirrhotic patients [5,6,7,8].

During inflammation, the balance between pro- and antioxidants could be disrupted, leading to increased oxidative stress [4]. The results include damage to cellular components and altered gene expression, which further lead to the development or progression of liver cirrhosis [9,10,11,12]. In general, restoring the balance between oxidative stress and antioxidant defense capacities, or even tilting it towards a stronger antioxidant defense capacity, could protect the liver from further damage and slow down or limit the disease progression. Glutathione (GSH) is a thiol and tripeptide molecule synthesized in the liver. The molecule is involved in several reactions of detoxifying electrophiles and scavenging free radicals, as well as in suppressing hydrogen peroxide formation [13]. Since liver is responsible for ≥90% of GSH turnover and interorgan GSH homeostasis [14,15], liver cirrhosis could not only affect the endogenous production and utilization of GSH, but also cause the overconsumption of this antioxidant nutrient. Cirrhotic patients have lower levels of plasma or erythrocyte GSH compared with normal subjects [11,16,17,18], with whole blood GSH concentrations dropping and rising during different stages of liver cirrhosis [12], or with erythrocyte GSH levels increasing in parallel with the severity of liver cirrhosis [19]. The reports on GSH status in patients with different severities of liver cirrhosis are, however, inconsistent among investigators.

In spite of GSH being the most abundant cellular thiol antioxidant, GSH may also have an anti-inflammatory role [20,21]. Although both increased inflammatory responses and the changes of GSH status are very likely associated with liver cirrhosis, it is unclear whether their actions in mediating liver damage occur independently or interactively with each other. The purpose of this study was to characterize inflammatory responses and GSH status of patients in different stages of liver cirrhosis, and compare them with healthy subjects. In addition, we assessed whether GSH status and inflammatory markers were independent or interactive with each other to mediate the severity of liver cirrhosis.

## 2. Subjects and Methods

### 2.1. Study Design and Sample Size Calculation

This was a cross-sectional study. Galicia-Moreno et al. [12] reported a significant correlation of 0.59 between levels of whole blood GSH and Child–Turcotte–Pugh scores in alcoholic patients with liver cirrhosis. Based on that report, the sample size was estimated for a significant correlation of 0.35 between plasma GSH concentration and Child–Turcotte–Pugh scores in patients with liver cirrhosis at a power of 80% and a two-sided test with an α of 0.05. The minimal sample size required was 62 patients. 

### 2.2. Subjects

Patients diagnosed with liver cirrhosis were recruited from the Division of General Surgery and Division of Gastroenterology and Hepatology of Taichung Veterans General Hospital, Taiwan. Diagnosis was made based on a variety of findings from clinical, histopathological, biochemical, and radiologic examinations. Biochemical parameters (i.e., serum albumin and bilirubin concentrations, prothrombin time), and subjective parameters (i.e., the degree of ascites and hepatic encephalopathy) were used to generate the score representing the severity of liver dysfunction. The severity scale is the following: class A (score 5–6), class B (score 7–9), and class C (score 10–15), which are derived from the Child–Turcotte–Pugh classification scheme [22]. Patients excluded were those with age ≤20 or ≥80 years, or with decompensated cirrhosis in clinical critical condition, pregnant or lactating, receiving chemotherapy, with diabetic, cardiovascular, renal, or chronic inflammatory diseases. Healthy subjects were recruited from the health management center of Taichung Veterans General Hospital, Taiwan. Subjects were excluded if they were ≤20 or ≥80 years of age, or had a history of chronic or metabolic diseases. All subjects had given signed informed consent. This study was approved by the Institutional Review Board of Taichung Veterans General Hospital (IRB TCVGH No. SF14261B). 

### 2.3. Data Collection and Biochemical Measurements

All personal data of subjects, like age, gender, and smoking and drinking habits, were first recorded. Their body height and weight were measured, and the body mass index (BMI) (kg/m^2^) was calculated. Systolic and diastolic blood pressures were measured after a resting period of ≥5 min. Since silymarin has been shown to increase intracellular GSH level [23], we recorded whether patients were prescribed to take silymarin as supportive elements of liver cirrhosis.

On the appointment day, fasting blood samples were drawn and collected in vacutainer tubes (Becton Dickinson, Rutherford, NJ, USA) either with or without anticoagulant. Serum samples were used to measure, with an automated biochemical analyzer, the white blood cell count, absolute neutrophil count, absolute lymphocyte count, absolute monocyte count, albumin, total bilirubin, alanine aminotransferases (ALT), and creatinine. The NLR was calculated (absolute neutrophil counts divided by the absolute lymphocyte count). Estimated glomerular filtration rate was calculated based on the KDIGO CKD work group [24]. Serum high-sensitivity C-reactive protein (hs-CRP) concentrations were determined using a particle-enhanced immunonephelometer equipped with an image analyzer. Plasma samples of the patients were used to determine the following: levels of malondialdehyde (MDA), GSH, glutathione disulfide (GSSG, oxidized form of GSH), activities of glutathione peroxidase (GSH-Px) and glutathione reductase (GSH-Rd), and Trolox equivalent antioxidant capacity (TEAC). Plasma samples of healthy subjects were used to determine levels of MDA, GSH, GSH-Px activity, and TEAC. Plasma MDA levels that reflect oxidative stress were measured with thiobarbituric acid reactive substances excited at a wavelength of 515 nm and an emission wavelength of 555 nm, using a fluorescence spectrophotometer (Jasco FP-2020 plus, Hachioji, Tokyo, Japan) [25]. Plasma GSH and GSSG were measured with the GSH and GSSG commercial kits (BioVision Incorporate, Milpitas, CA, USA). Activities of plasma GSH-Px were determined with the GSH-Px commercial kit (Cayman Chemical Company, Ann Arbor, MI, USA). The plasma GSH-Rd activity was assessed according to the method of Carlberg & Mannervik [26]. TEAC was estimated according to the method of Erel [27]. All measurements were done in duplicates.

### 2.4. Statistical Analysis

Data analyses were done using the SAS statistical software package (version 9.3; Statistical Analysis System Institute Inc., Cary, NC, USA). The Shapiro–Wilk test was used for the normality test of sample distributions. Values presented in the text are means ± standard deviation. The median value and interquartile range were added in the parentheses for non-normally distributed values. Demographic characteristics and biochemical data were compared using a one-way analysis of variance or Kruskal–Wallis one-way analysis of variance of ranks, to determine significant differences across groups. Chi-square or Fisher’s exact tests were used in the analyses of categorical variables. Multiple linear regression analyses, with the Child–Turcotte–Pugh score as a dependent variable, were used to assess the association of severity of liver cirrhosis with either individual or combined inflammatory indicators, GSH, GSSH, and GSH/GSSG ratio, after adjusting for potential confounders (i.e., age, gender, BMI, smoking and drinking habits, hepatitis, and silymarin use). Statistical significance was set at *p* < 0.05 (two-sided).

## 3. Results

Data from 75 patients with liver cirrhosis and 110 healthy subjects were analyzed. As the numbers of patients were too small in both class B and C (9 in class B, and 3 in class C), we chose to combine them into one group (class B–C) and the remaining patients (63) in another group (class A) for comparisons. There were 16 patients (25.4%) in class A group, and 3 patients (25%) in class B–C group were taking oral capsules of silymarin. Characteristics of patients and healthy subjects are shown in Table 1. Patients in the class A group were older, had higher systolic blood pressure, serum ALT and creatinine levels compared with healthy subjects. Patients in class B–C group had the lowest serum albumin, and the highest serum total bilirubin levels, when compared to patients in class A group and healthy subjects. Table 2 shows the levels of inflammatory markers and indicators of oxidative stress and antioxidant capacity in patients and healthy subjects. Patients in group class B–C had the highest levels of hs-CRP compared with those of patients in class A and healthy subjects. Patients in class B–C had greater oxidative stress (i.e., in terms of plasma MDA levels) when compared to patients in class A and healthy subjects. Patients in class A had significantly higher levels of GSH, GSSG, GSH/GSSG ratio, and higher GSH-Px activity when compared to patients in class B–C or healthy subjects. 

Table 3 shows the results from multiple linear regressions regarding the individual or combined effects of NLR, hs-CRP, GSH, GSSG, and GSH/GSSG ratio on the severity of liver cirrhosis. The NLR ratio and GSSG level were not associated with the Child–Turotte–Pugh scores after adjustment for potential confounders. Serum hs-CRP levels were significantly and positively associated with the scores, while GSH levels were significantly but negatively associated with the scores again after adjusting for basic potential confounders and each other. We found significant interactions between levels of serum hs-CRP and plasma GSH (*p* = 0.016), but we found no interactions between NLR and GSH. Results suggested that hs-CRP and GSH had interacted with each other in the association with Child–Turotte–Pugh scores.

## 4. Discussion

Our findings indicated that inflammatory responses were elevated in patients with more severe liver cirrhosis. However, only CRP, but not NLR, was found here to be associated with Child–Turotte–Pugh scores. NLR may not faithfully reflect the inflammatory status of the liver, since lymphomononuclear cells are mainly involved in hepatic inflammation in those patients with chronic hepatitis and cirrhosis [28,29]. Tanoglu & Karagoz [30] have also cautioned that several clinical conditions may affect NLR levels. Therefore, in agreement with Tanoglu & Karagoz [30], the NLR level alone, without information from other contributing variables, might not provide accurate prognostic estimates of liver cirrhosis. On the other hand, while CRP is synthesized in the liver during acute phase of inflammation (as a response to cytokine productions), its concentration is not altered in impaired livers [31]. Since inflammatory responses and antioxidant capacities have not been examined in the previous studies regarding their possible interactions [5,7], the synergistic effects of CRP and GSH on the severity of liver cirrhosis cannot be ignored.

Although in the clinical setting, hepatic GSH concentration per se cannot be determined, plasma GSH concentration, which is measureable, could reflect, to some extent, its intrahepatic levels [32]. The ratio of reduced and oxidized forms of GSH (GSH/GSSG) in plasma is used to evaluate cellular oxidative stress [33], and the change from GSH to GSSG reduces cell proliferation and increases apoptosis [34]. We thus determined plasma GSH and GSSG concentrations, and calculate plasma GSH/GSSG ratio to represent intrahepatic levels and cellular oxidative stress, respectively. Previous studies indicated that plasma GSH concentration was similar, but plasma GSSG concentration was significantly higher in chronic hepatitis C patients with cirrhosis [17], and patients with alcoholic liver disease [35], when compared to the levels of healthy controls. Erythrocyte GSH concentrations are higher in class B cirrhotic patients than those in class A and healthy controls [19]. However, both the GSH concentration and GSH-Px activity, as found in the present study, were ~1.5 times higher in the class A group, and dropped to a level similar to that in the class B–C group, when compared to healthy subjects. This finding implied that patients with mild liver dysfunction increased their GSH turnover to cope with an increasing oxidative stress. Although we did not measure plasma GSH-Rd activity of healthy subjects and no previous published results can be compared, plasma GSH-Rd activity seemed to have an opposite performance with GSH-Px activity in class A and B–C group. It remains unclear whether GSH synthesis is unaltered or impaired with the further progression of liver cirrhosis. Among enzymes controlling for the utilization of GSH, γ-glutamyltransferase (also called γ-glutamyltranspeptidase, GGT) is a liver canalicular enzyme responsible for transporting GSH across cell membranes to provide the cells with the amino acids necessary for the de novo synthesis of GSH, and its defect might affect antioxidant defense, detoxification, and inflammation processes in the pathology of disease [36]. Since increased or decreased GGT activity has been shown to affect GSH utilization in patients with different kinds of liver diseases [36], we could not rule out the possibility that abnormal GGT activity might affect GSH utilization in our cirrhotic patients. However, abnormal GGT activity could be the functional result of liver cirrhosis, rather than the causal factor. Since GGT activity was not measured in the present study, the possible association of GGT activity with plasma GSH levels in cirrhotic patients requires further investigation. 

The association between GSH status and cirrhotic severity has some pathogenic significances. However, their relationship remains controversial and unconfirmed. A previous study showed that whole blood GSH concentration is positively associated with Child–Pugh scores in patients with alcoholic liver cirrhosis [12]. In our study, however, plasma GSH concentration was negatively associated with the Child–Turcotte–Pugh scores. Another study also found that erythrocyte GSH concentration is negatively associated with the stages of hepatic fibrosis in patients with chronic hepatitis C [37]. The discrepancy in results across studies could be due to heterogeneity in the sampled patients, and different causative factors involved in cirrhosis. The inflammation that resulted from viral insult occurs mainly in the periportal hepatocytes (zone 1 of acinus), while that from toxins is in the perivenous hepatocytes (zone 3 of acinus) [38]. The intracellular GSH levels appear to vary across different zones of hepatic acinus. The intracellular GSH concentration and the activity of synthesis of zone 1 hepatocytes are higher than those of zone 3 hepatocytes [39]. Since over half of our patients had chronic viral hepatitis, it is reasonable to speculate that the zone 1 hepatocytes, being injured more extensively, would be responsible for the lower GSH levels of class B–C cirrhotic patients. In agreement with previous studies [10,37], the gradual loss of GSH status in liver cirrhosis might be related to reduced hepatic GSH efflux or impaired GSH synthesis as the disease advances.

The uniqueness of this study is the simultaneous examination of the association of inflammatory responses and antioxidant capacity with the severity of liver cirrhosis. Four limitations of the study should be pointed out. The first is that the cases of liver cirrhosis were not evenly distributed across the severity scale. For example, due to the small numbers of patients, those in the Child–Turcotte–Pugh scores B and C had to be combined into a single group for analyses, and thus, scores, rather than classes, were practically used to determine the association of factors with the severity of the disease. Therefore, our results should be compared in more evenly distributed samples and with more patients recruited for analyses. The second is that all biochemical measurements were made at single time points. Results might not truly reflect the associations we were studying. Thirdly, GSSG levels and GSH-Rd activities were not measured in the healthy subjects due to limited volume of their blood samples we had obtained. Therefore, the picture of GSH metabolism could not be fully determined as we would have wished for better comparisons. Finally, a larger sample size may offer more statistical power to deal with the association between the severity of liver cirrhosis and inflammatory indicators and GSH status.

## 5. Conclusions

CRP and GSH had exerted synergistic effects in the association with the severity of liver cirrhosis. The decreased GSH antioxidant capacity and increased inflammatory responses should be considered by clinicians in the treatment of liver cirrhotic patients.

## Figures and Tables

**Table 1:** Demographic and biochemical characteristics of patients with liver cirrhosis and healthy subjects.

Characteristics	Liver Cirrhosis		Healthy Subjects (*n* = 110)
	Class A (*n* = 63)	Class B–C (*n* = 12)	
Age (y)	59.7 ± 9.3 ^a^	57.6 ± 12.6 ^a,b^	50.4 ± 7.9 ^b^
Gender (Male/Female)	49/14	9/3	48/62
Height (cm)	162.5 ± 8.6 (163.5, 158.4–168.9)	167.7 ± 9.5	163.5 ± 7.9
Weight (kg)	65.9 ± 10.5 ^a,b^	76.0 ± 15.1 ^a^	62.9 ± 11.8 ^b^ (61.2, 53.6–71.3)
Body mass index (kg/m^2^)	25.0 ± 3.6 ^a^	27.1 ± 5.8 ^a^	23.4 ± 3.4 ^b^ (22.8, 20.9–25.8)
Blood pressure (mmHg)			
Systolic	131.7 ± 18.1 ^a^	127.3 ± 22.9 ^a,b^	117.0 ± 17.6 ^b^
Diastolic	79.4 ± 12.6	76.8 ± 15.6	75.0 ± 11.8
Serum albumin (g/dL)	4.2 ± 0.5 ^b^ (4.3, 3.9–4.6)	2.9 ± 0.4 ^c^	4.5 ± 0.2 ^a^
Serum creatinine (mg/dL)	1.0 ± 0.5 ^a^ (0.9, 0.8–1.0)	1.2 ± 0.7 ^a^ (0.9, 0.8–1.4)	0.8 ± 0.2 ^b^ (0.8, 0.6–0.9)
eGFR (mL/min/1.73 m^2^)	81.5 ± 26.2 ^b^	89.1 ± 47.7 ^a,b^	98.0 ± 23.4 ^a^ (94.2, 80.4–112.2)
Serum ALT (U/L)	57.4 ± 59.0 ^a^ (38.0, 26–60.3)	43.9 ± 31.1 ^a^ (36.5, 23.0–53.0)	22.6 ± 7.4 ^b^ (22.0, 16.0–27.0)
Serum total bilirubin (mg/dL)	1.0 ± 0.6 ^a^ (0.9, 0.6–1.3)	4.5 ± 3.0 ^b^	0.9 ± 0.5 ^a^ (0.9, 0.7–1.1)
Hepatitis (*n*, %)			
Hepatitis B	21, 33.3%	0	-
Hepatitis C	15, 23.8%	2, 16.7%	-
Co-hepatitis B and C	1, 1.6%	0	-
Current smoking habit (*n*, %)	17, 27.0%	6, 50%	17, 15.5%
Current drinking habit (*n*, %)	7, 11.1%	1, 8.3%	25, 22.7%

Values are means ± standard deviation. The median value and interquartile range are in the parentheses for non-normally distributed values. ALT, alanine aminotransferase; eGFR, estimated glomerular filtration rate. ^a,b,c^ Values with different superscript letter are significantly different among groups; *p* < 0.05.

**Table 2:** Inflammatory markers and oxidative stress indicators in patients with liver cirrhosis and healthy subjects.

Parameters	Liver Cirrhosis		Healthy Subjects (*n* = 110)
	Class A (*n* = 63)	Class B–C (*n* = 12)	
*Inflammatory markers*			
White blood cell (cells/mm^3^)	5316.83 ± 2039.96 (5120.0, 3945.0–6550.0)	5216.00 ± 1953.31	5210.00 ± 1599.77 (5100.0, 4300.0–5700.0)
Absolute neutrophil count (cells/mm^3^)	3410.07 ± 1556.4 (3012.68, 2289.6–4175.35)	3281.46 ± 1850.12 (3165.50, 2514.5–3411.0)	2971.63 ± 1170.50 (2823.45, 2332.0–3302.0)
Absolute lymphocyte count (cells/mm^3^)	1413.51 ± 633.21	1315.45 ± 614.77	1705.98 ± 577.77 (1657.75, 1328.1–1947.0)
Neutrophil/Lymphocyte ratio	2.90 ± 1.75 ^a^ (2.35, 1.63–3.69)	2.50 ± 1.45 ^a,b^	1.86 ± 0.85 ^b^ (1.69, 1.32–2.17)
Absolute monocyte count (cell/mm^3^)	362.83 ± 170.03 (311.14, 239.12–471.3)	513.86 ± 255.30	376.53 ± 196.16 (340.4, 283.75–420.60)
hs-CRP (mg/dL)	0.99 ± 3.79 ^b^ (0.10, 0.04–0.26)	2.35 ± 4.77 ^a^ (0.75, 0.43–1.86)	0.06 ± 0.07 ^c^ (0.03, 0.01–0.07)
*Indicators of oxidative stress and antioxidant capacity*			
Malondialdehyde (μmol/L)	0.79 ± 0.19 ^b^ (0.76, 0.67–0.90)	0.93 ± 0.20 ^a^	0.85 ± 0.19 ^a,b^ (0.81, 0.71–0.96)
Glutathione (μmol/L)	77.76 ± 25.41 ^a^	42.88 ± 27.47 ^b^ (42.03,29.98–47.30)	44.69 ± 17.38 ^b^
Glutathione disulfide (μmol/L)	667.03 ± 70.96 ^a^	601.22 ± 87.64 ^b^	-
GSH/GSSG ratio	0.12 ± 0.04 ^a^	0.07 ± 0.04 ^b^	-
GSH-Px (nmol/mL/min)	205.17 ± 68.33 ^a^ (206.30, 159.18–237.50)	183.80 ± 109.49 ^a,b^	148.05 ± 36.57 ^b^ (140.08, 119.71–165.55)
GSH-Rd (nmol/mL/min)	76.73 ± 33.44 ^b^ (71.58, 61.84–83.58)	87.87 ± 23.51 ^a^ (83.59, 77.48–89.79)	-
TEAC (μmol/L)	4227.95 ± 640.79 (4188.40, 3886.14–4534.17)	4041.76 ± 819.51	4290.79 ± 468.35 (4290.93, 3974.34–4590.63)

Values are means ± standard deviation. The median value and interquartile range are in the parentheses for non-normally distributed values. hs-CRP, high sensitivity C-reactive protein; GSH, glutathione; GSSG, glutathione disulfide; TEAC, Trolox equivalent antioxidant capacity. ^a,b,c^ Values with different superscript letter are significantly different among groups; *p* < 0.05.

**Table 3:** Multiple linear regression analysis with Child–Turcotte–Pugh score as the dependent variable in patients with liver cirrhosis after adjusting for potential confounders.

Parameters	Covariate Model	*β* (Standard Error)	*p* Value
NLR	Confounders ^1^	0.095 (0.084)	0.264
	GSH + confounders	0.101 (0.077)	0.197
	GSH + NLR × GSH + confounders	−0.264 (0.221)	0.237
hs-CRP	Confounders	0.074 (0.036)	0.065
	GSH + confounders	0.099 (0.035)	0.007
	GSH + hs-CRP × GSH + confounders	−0.275 (0.154)	0.079
GSH	Confounders	−0.014 (0.005)	0.007
	NLR + confounders	−0.016 (0.004)	<0.001
	hs-CRP + confounders	−0.017 (0.005)	<0.001
	NLR + NLR × GSH + confounders	−0.030 (0.009)	0.002
	hs-CRP + hs-CRP × GSH + confounders	−0.021 (0.005)	<0.001
NLR × GSH	NLR + GSH + confounders	0.005 (0.003)	0.084
hs-CRP × GSH	hs-CRP + GSH + confounders	0.004 (0.001)	0.016
GSSG	Confounders	−0.002 (0.002)	0.272
GSH/GSSG ratio	Confounders	−9.741 (3.468)	0.007

*n* = 75. *β*, regression coefficient. NLR, neutrophil-to-lymphocyte ratio; hs-CRP, high sensitivity C-reactive protein; GSH, glutathione; GSSG, glutathione disulfide. ^1^ Adjusted for age, sex, body mass index, smoking and drinking status, hepatitis, and silymarin use.

## Abbreviations

The following abbreviations are used in this manuscript:

ALT	alanine aminotransferases
BMI	body mass index
hs-CRP	high-sensitivity C-reactive protein
GSH	glutathione
GSH-Px	glutathione peroxidase
GSH-Rd	glutathione reductase
GSSG	oxidized glutathione
MDA	malondialdehyde
NLR	neutrophil-to-lymphocyte ratio
TEAC	Trolox equivalent antioxidant capacity
